# Gene polymorphisms and expression levels of interleukin-6 and interleukin-10 in lumbar disc disease: a meta-analysis and immunohistochemical study

**DOI:** 10.1186/s13018-020-01588-8

**Published:** 2020-02-18

**Authors:** Yewen Guan, Siting Wang, Jiaqi Wang, Dihua Meng, Huihong Wu, Qingjun Wei, Hua Jiang

**Affiliations:** 1 Division of Spine Surgery, The First Affiliated Hospital of Guangxi Medical University, No.6 Shuangyong Road, Nanning, 530021 China; 2 Department of Orthopaedic Surgery, The First Affiliated Hospital of Guangxi Medical University, No.6 Shuangyong Road, Nanning, 530021 China

**Keywords:** IL-6, IL-10, Lumbar disc disease, Meta-analysis, Polymorphisms

## Abstract

**Background:**

To investigate the association between interleukin-6 (IL-6) (rs1800795, rs1800796, rs1800797, rs13306435, rs2069849) and interleukin-10 (IL-10) (rs1800871, rs1800896) gene polymorphisms, expression levels, and lumbar disc disease (LDD).

**Methods:**

We conducted a literature research on PubMed, Embase, Web of Science, Cochrane Library, and China National Knowledge Infrastructure (CNKI) until February 28, 2019. We included all case-control studies about the association between IL-6 and IL-10 gene polymorphisms and LDD. The odds ratio (OR) and 95% confidence interval (CI) were calculated to estimate the strength of association. Statistical analysis was conducted by Review Manager (RevMan) 5.3 software. Furthermore, immunohistochemistry (IHC) and RT-PCR were performed to evaluate IL-6 and IL-10 expressions in the normal and degenerated disc.

**Results:**

A total of 6 studies, involving 1456 cases and 1611 controls, were included in this meta-analysis. G alleles of rs1800795 and rs1800797 in the IL-6 gene were significantly associated with LDD (rs1800795: G vs. C, OR = 1.38, 95% CI = 1.16–1.64, *P* = 0.0002; rs1800797: G vs. A, OR = 1.35, 95% CI = 1.14–1.61, *P* = 0.0006). Begg’s funnel plot and Egger’s tests did not show any evidence of publication bias. IL-6 expression and IL-6 mRNA levels were significantly increased in the degenerated disc compared with those in the normal disc (IL-6 immunopositive cells, 73.68 ± 10.99% vs. 37.23 ± 6.42%, *P* < 0.001).

**Conclusions:**

IL-6 gene polymorphisms (rs1800795 and rs1800797) were significantly associated with susceptibility to LDD. A high expression level of IL-6 may be an important risk factor for LDD.

## Introduction

Low back pain (LBP) is a common musculoskeletal disorder affecting the general and athletic populations. Over 80% of the people will suffer from LBP at least one episode in their lifetime [1]. LBP is associated with significant disability and a reduction in quality of life, thus posing a high burden to society [2, 3]. Lumbar disc degeneration (LDD) is one of the major causes of LBP [4]. The etiology of LDD is unclear, but various genetic and environmental factors have been identified [5–7]. Recently, increasing evidence suggested that several genes, such as interleukins (ILs), collagen IX, and matrix metalloproteinases (MMPs), play a vital role in the initiation and progression of LDD [8, 9].

Over the past few years, the associations of IL-6 and IL-10 gene polymorphisms with LDD have been investigated in a multitude of studies [10–15]. In 2005, Noponen-Hietala et al. [10] first studied the association between IL-6 and IL-10 gene polymorphisms (rs1800795, rs1800796, rs1800797, rs13306435, rs2069849, and rs1800896) and LDD risk. The result showed that rs13306435 of IL-6 gene was associated with LDD in the Finnish population. Subsequently, other studies contradicted the previous study stating that rs13306435 was not relevant to LDD, but other SNPs (rs1800795, rs1800796, and rs1800797) in the IL-6 gene were relevant to LDD [11, 12, 14, 15]. Moreover, several studies demonstrated that IL-10 gene polymorphisms (rs1800871, rs1800896) were associated with LDD susceptibility [13, 15]. However, the results were not confirmed by the following studies [14]. Despite extensive research in this field, the results were generally inconsistent and inconclusive. In view of these considerations, we undertook a meta-analysis to investigate the association between IL-6 and IL-10 gene polymorphisms and LDD. The SNPs rs1800795, rs1800796, and rs1800797 were located in the IL-6 gene promoter, and rs1800871 and rs1800896 were in the promoter area of the IL-10 gene. These functional SNPs could increase the transcriptional activity of IL-6 and IL-10 promoter, leading to the upregulation of IL-6 and IL-10 in stress or infection [16, 17]. In addition, polymorphisms (rs13306435 and rs2069849) in the IL-6 gene exon have been reported to be associated with different profiles of plasma IL-6 response to immunization [18]. As polymorphisms of IL-6 and IL-10 gene may alter IL-6 and IL-10 expressions [19], we used immunohistochemistry (IHC) and RT-PCR to evaluate IL-6 and IL-10 expression levels in intervertebral disc between the LDD patients and the control subjects.

## Materials and methods

### Strategy for literature search

This meta-analysis was conducted in accordance with a prespecified protocol registered with PROSPERO International Prospective Register of Systematic Reviews (CRD42019124118). Two authors (YW Guan and ST Wang) searched all major databases up to February 28, 2019, including PubMed, Embase, Web of Science, Cochrane Library, and China National Knowledge Infrastructure (CNKI). The following keywords were used for searching: (“LDD” OR “lumbar disc disease” OR “lumbar disc degeneration” OR “intervertebral disc degeneration”) AND (“IL-6” or “interleukin-6” OR “IL-10” OR “interleukin-10”) AND (“SNP” OR “polymorphisms”). In addition, we checked the reference lists of the included studies for further relevant literature. No publication date or language restrictions were implemented. The authors strictly adhered to the Preferred Reporting Items for Systematic Review and Meta-Analysis Protocols (PRISMA-P) guidelines [20] throughout the study.

### Inclusion and exclusion criteria

To include in all the eligible articles and exclude ineligible articles, the inclusion and exclusion criteria were established. The studies were included in the review if they (1) recruited the experimental subjects which were diagnosed as LDD by clinical or/and radiological examination, (2) were cohort-based or case-control studies that assessed the associations of IL-6 and IL-10 gene polymorphisms with LDD, (3) evaluated the genotype of the control group and conformed to the Hardy-Weinberg balance, and (4) calculated the odds ratios (OR) to assess the association. Exclusion criteria were (1) repeated publications; (2) conference abstracts, letters to editor, and unpublished studies; and (3) incomplete data on allele and genotype frequencies. Articles published in languages other than English were translated. Based on the inclusion and exclusion criteria, two authors (YW Guan and ST Wang) independently screened the titles and abstracts of references and obtained the full text for reference. Disagreements between two authors were resolved by discussion, and if necessary, a third author (H Jiang) was consulted.

### Data extraction

Based on a standardized form, two authors (YW Guan and ST Wang) independently extracted data on outcomes for each study. For disagreements, a consensus was reached by a third author (H Jiang). Extracted parameters included the following: (1) first author, (2) publication year, (3) study population (country, ethnicity), (4) study design, (5) numbers of cases and controls, (6) characteristics of participants (age and gender), (7) diagnostic criteria, (8) source of controls, (9) allele or/and genotype frequencies, and (10) results of Hardy-Weinberg equilibrium (HWE) test. Hardy-Weinberg equilibrium was checked in study controls using the *χ*^2^ goodness-of-fit test as a quantitative assessment for potential selection bias and confounding.

### Methodological quality

Methodological quality of studies was independently assessed by two investigators according to a quality evaluation form (Critical Appraisal Skills Programme for case- control study, CASP), which is based on ten questions associated with information given by each article [21]. Each question has three degrees behind, “yes” (scored 2), “cannot tell” (scored 1), and “no” (scored 0). The maximum total score is 20. Studies could be divided into three grades: grade A (high quality, scored 15–20), grade B (medium quality, scored 8–14), and grade C (low quality, scored 0–7).

### Study population

Based on our previous study [22], we collected degenerative disc tissues (*n* = 34) and normal disc tissues (*n* = 21) from the LDD patients and the control subjects. LDD patients were diagnosed with lumbar disc herniation by physical examination and MRI scan. The control subjects were the patients with traumatic lumbar vertebral fracture, who had no history of low back pain. Based on Schneiderman’s classification [23], MRI evaluation showed no significant disc damage and degeneration before surgery (Schneiderman’s classification, grade 1, 19 cases; grade 2, 2 cases). These disc samples were used to evaluate IL-6 and IL-10 expressions via IHC and RT-PCR. This study was approved by the institute’s ethics committee for human studies (2018-KY-NSFC-025). Informed consent was obtained from all the participants in this study.

### Immunohistochemistry

The intervertebral disc tissue was fixed in 10% neutral buffered formalin within 1 h after surgical excision and was embedded in paraffin for serial sectioning at 3 μm. After routinely xylene dewaxing and gradient ethanol hydration, the sections were incubated in 0.01 mol/L citrate buffer (pH = 6). The sections were immersed in 3% hydrogen peroxide for 10 min and subsequently rinsed three times with PBS buffer solution for 3 min each time at room temperature. Nonspecific binding was blocked with 3% normal goat serum in a phosphate-buffered saline solution (pH = 7.4) for 15 min at room temperature. Primary antibody (polyclonal rabbit anti-IL-6/anti-IL-10, ab6672/ab34843, Abcam, Cambridge, UK) was diluted at 1:400 and incubated at 4 °C overnight. After washing, it was followed by secondary antibody (Biotin-labeled Goat Anti-Rabbit IgG, SP-9001, ZYMED, USA) for 15 min at room temperature. Then, the sections were incubated in Horseradish Peroxidase Streptavidin for 15 min. DAB reagent was added to the section that was examined by microscope and incubated for 10 min at room temperature. Sections were counterstained with hematoxylin for 10 min, dehydrated through several baths of graded hydrochloric alcohol and xylene dehydration, and then mounted using Neutral Balsam. The results of immunohistochemistry (IHC) were obtained using an Olympus BX43 upright microscope (Olympus Optical, Tokyo, Japan). In addition, fresh intervertebral disc tissues were collected to obtain the total RNA. The total mRNA was extracted with TRIzol (Invitrogen Life Technologies, CA, USA). One microgram of the total RNA was used to synthesize complementary DNA (cDNA) using an iScript cDNA Synthesis kit (Quanta Biosciences, MD, USA). Subsequently, real-time PCR amplification was performed using specific primers of target genes and a SYBR Green real-time PCR kit (Quanta Biosciences, MD, USA). The primer sequences were selected according to previous study [24].

### Statistical analysis

Odds ratio (OR) and 95% confident intervals (CI) were used to assess the strength of associations between IL-6 and IL-10 gene polymorphisms with LDD susceptibility which were conducted under five genetic models. Chi-square-based *Q* test and *I*^2^ test were used to check the heterogeneity among the included studies. A fixed-effect model was used while no heterogeneity existed (*P* > 0.10, *I*^2^ < 50%). Otherwise, a random-effects model was used (*P* < 0.10, *I*^2^ > 50%). For comparisons which have significant heterogeneity, we have performed a sensitive analysis to evaluate the effect of one study on the pooled OR. The Hardy-Weinberg equilibrium of controls was calculated using the HWE Version 1.20 program. Potential publication bias was assessed using Begg’s funnel plot and Egger’s tests. IHC and RT-PCR data were presented as the mean ± standard error. Statistical difference between the two groups was evaluated using unpaired Student’s *t* test. Statistical significance was set at *P* < 0.05. Data analysis was conducted using SPSS 20.0 statistical software (SPSS Inc., Chicago, IL, USA) and Review Manager 5.31 (Nordic Cochrane Center: http://ims.cochrane.org/revman/ download).

## Results

### Characteristics of included studies

**Fig. 1:**
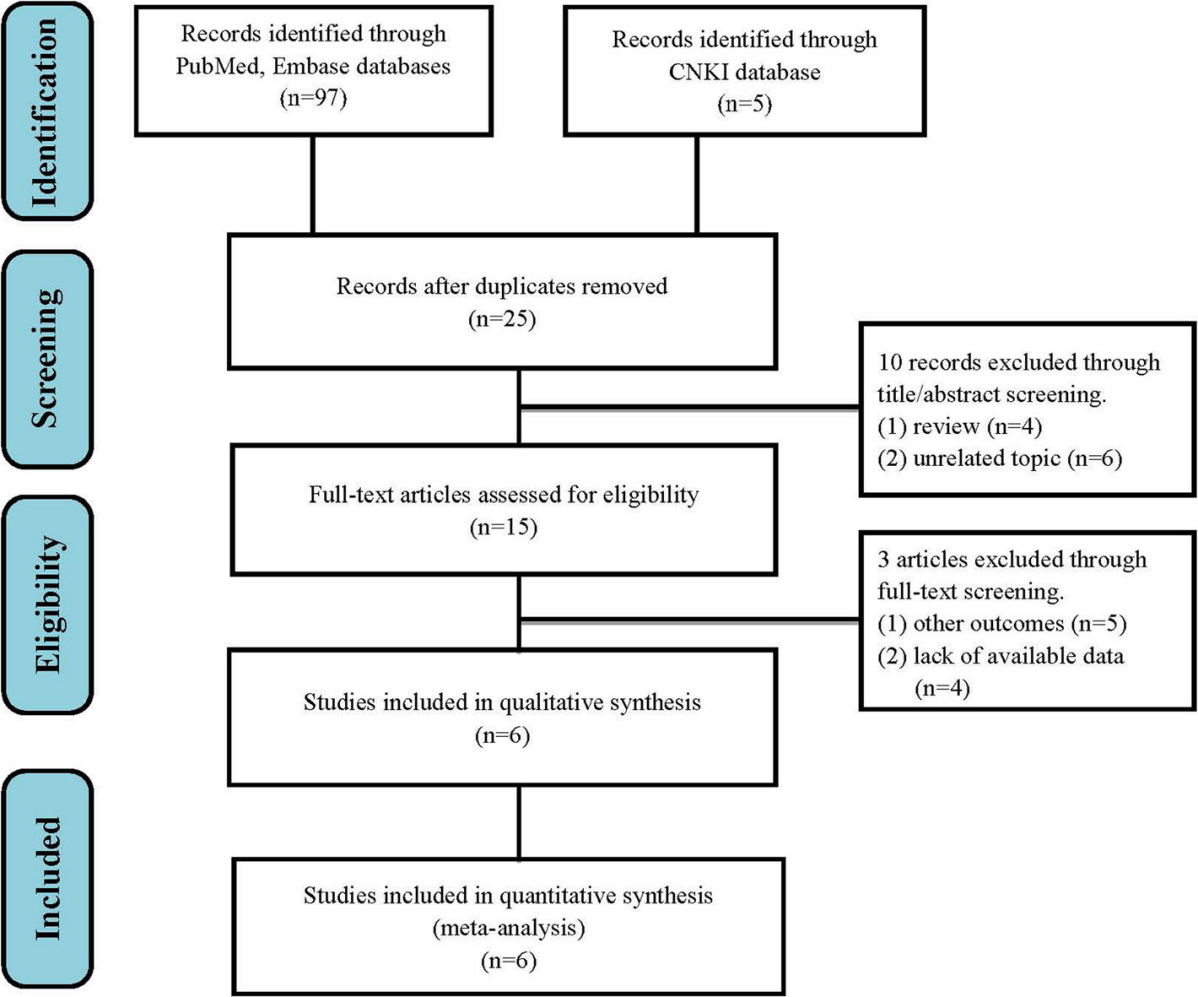
Literature search strategy and selection of articles. A total of 102 articles were selected for the meta-analysis by browsing the databases PubMed, Embase, and CNKI, of which 77 articles were excluded after reviewing the title and abstract and 10 articles were excluded after reviewing the full publications. Finally, a total of 6 studies were considered for the meta-analysis

The flow chart for the identification of the studies is presented in Fig. 1. A total of 6 studies were finally identified, including 1456 cases and 1611 controls (rs1800795: 4 studies, 638 cases, and 879 controls; rs1800796: 5 studies, 1136 cases, and 1342 controls; rs1800797: 3 studies, 371 cases, and 579 controls; rs2069849: 2 studies, 221 cases, and 333 controls; rs13306435: 2 studies, 221 cases, and 333 controls; rs1800896: 3 studies, 742 cases, and 748 controls; rs1800871: 2 studies, 587 cases, and 569 controls). The main characteristics of included studies are presented in Table 1 and Table 2. For quality assessment, all selected studies are categorized as grade A according to scores ranging from 15 to 19 (Table 1).

**Table 1:** Characteristics of the case-control studies included in meta-analysis

First author	Year	Country	Ethnicity	Age (year, mean ± sd)		Number cases/controls	Diagnostic criteria	Control group	Outcome measure (SNP genotyping)	HWE for controls	CASP
				Cases	Controls						
Noponen-Hietala [10]	2005	Finland	Caucasian	44 ± 13	39 ± 13	155/179	MRI	NM	rs1800795, rs1800796, rs1800797, rs2069849, rs1800896	0.97	16
Eskola [11]	2010	Denmark	Caucasian	13.1 ± 0.4	13.1 ± 0.4	66/154	MRI	Patients without LDD	rs1800795, rs1800796, rs1800797, rs2069849, rs13306435	0.74	17
Kelempisioti [12]	2011	Finland	Caucasian	19	19	150/246	MRI	Patients without LDD	rs1800795, rs1800796, rs1800797, rs13306435	0.92	17
Lin [13]	2011	China	Asian	46.3 ± 8.4	46.5 ± 9.7	320/269	CT, MRI	NM	rs1800896, rs1800871	0.28	19
Huang [14]	2017	China	Asian	44.05 ± 9.02	41.85 ± 11.02	267/300	MRI	Patients without spine-related problems	rs1800795, rs1800796, rs1800896, rs1800871	0.93	18
Zhu [15]	2017	China	Asian	50.27 ± 12.53	50.65 ± 11.79	498/463	MRI	Patients without LDD	rs1800796	0.30	15

*NM* not mentioned, *MRI* magnetic resonance imaging, *CT* computerized tomography, *CASP* Critical Appraisal Skills Programme

**Table 2:** Distribution of genotypes and alleles of IL-6 and IL-10 gene polymorphisms in LDD patients and controls

Study			Case group					Control group					HWE for control
Author	Year	Ethnicity	11	12	22	1	2	11	12	22	1	2	
rs1800795 (G vs. C)													
Noponen-Hietala [10]	2005	European	47	77	31	171	139	37	89	53	163	195	0.97
Eskola [11]	2010	European	19	33	14	71	61	37	79	38	153	155	0.74
Kelempisioti [13]	2011	European	35	77	38	147	153	39	117	90	196	296	0.92
Huang [14]	2017	Asian	264	3	0	531	3	297	3	0	597	3	0.93
rs1800796 (C vs. G)													
Noponen-Hietala [10]	2005	European	0	5	150	5	305	0	15	164	16	342	0.55
Eskola [11]	2010	European	1	10	55	12	120	1	12	141	14	294	0.20
Kelempisioti [13]	2011	European	0	9	141	9	291	0	15	231	15	477	0.62
Huang [14]	2017	Asian	138	113	16	389	145	193	102	5	488	112	0.03
Zhu [15]	2017	Asian	55	233	210	653	343	239	181	43	659	267	0.30
rs1800797 (G vs. A)													
Noponen-Hietala [10]	2005	European	48	76	31	172	138	37	89	53	163	195	0.97
Eskola [11]	2010	European	17	34	15	68	64	37	79	38	153	155	0.74
Kelempisioti [13]	2011	European	34	79	37	147	153	43	116	87	201	291	0.68
rs13306435 (A vs. T)													
Noponen-Hietala [10]	2005	European	0	16	139	16	294	0	4	175	4	354	0.87
Eskola [11]	2010	European	0	1	65	1	131	0	4	150	4	304	0.87
rs2069849 (T vs. C)													
Noponen-Hietala [10]	2005	European	0	8	147	8	302	1	17	161	19	339	0.46
Eskola [11]	2010	European	0	3	63	3	129	0	11	143	11	297	0.64
rs1800871 (A vs. C)													
Lin [13]	2011	Asian	150	113	57	413	227	89	119	61	297	241	0.08
Huang [14]	2017	Asian	91	143	33	325	209	119	153	28	391	209	0.03
rs1800896 (G vs. A)													
Noponen-Hietala [10]	2005	European	32	76	47	140	170	34	88	57	156	202	0.99
Lin [13]	2011	Asian	118	113	89	349	291	122	93	54	337	201	< 0.01
Huang [14]	2017	Asian	0	70	197	70	464	0	35	265	35	565	0.28

11, 12, and 22 indicate GG GC CC for rs1800795, CC CG GG for rs1800796, GG GA AA for rs1800797, AA AT TT for rs13306435, TT TC CC for rs209849, AA AC CC for rs1800871, and GG GA AA for rs1800896 respectively

### IL-6 and IL-10 gene polymorphisms and LDD susceptibility

**Fig. 2:**
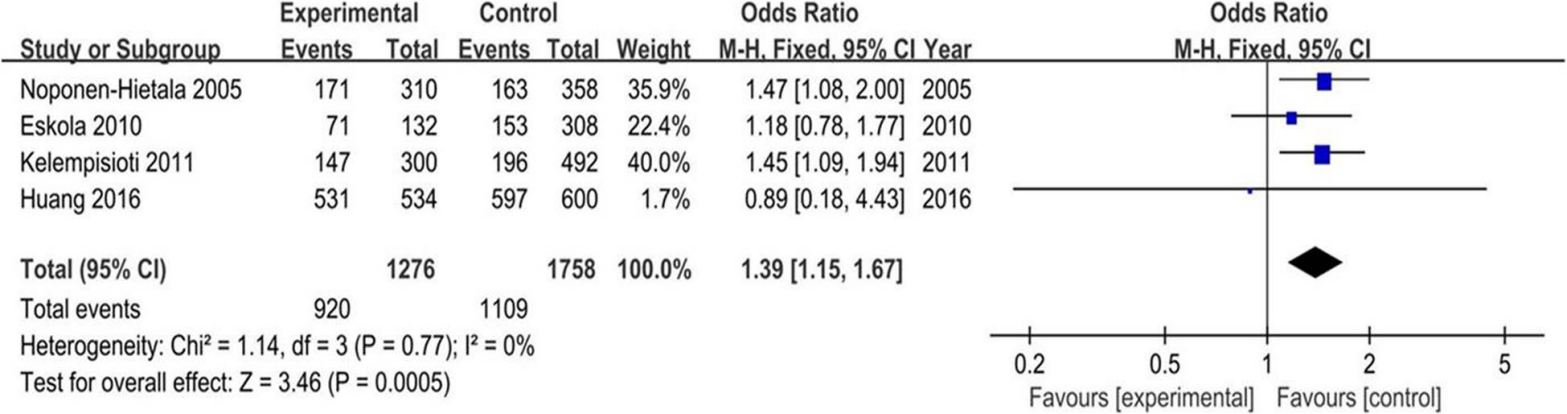
Forest plot of association between IL-6 gene polymorphism (rs1800795) and LDD risk under allelic contract model (G vs. C). There was a significant association between rs1800795 and LDD risk (G vs. C, OR 1.39, 95% CI 1.15–1.67, *P* = 0.0005). OR, odds ratio; CI, confidence interval

**Fig. 3:**
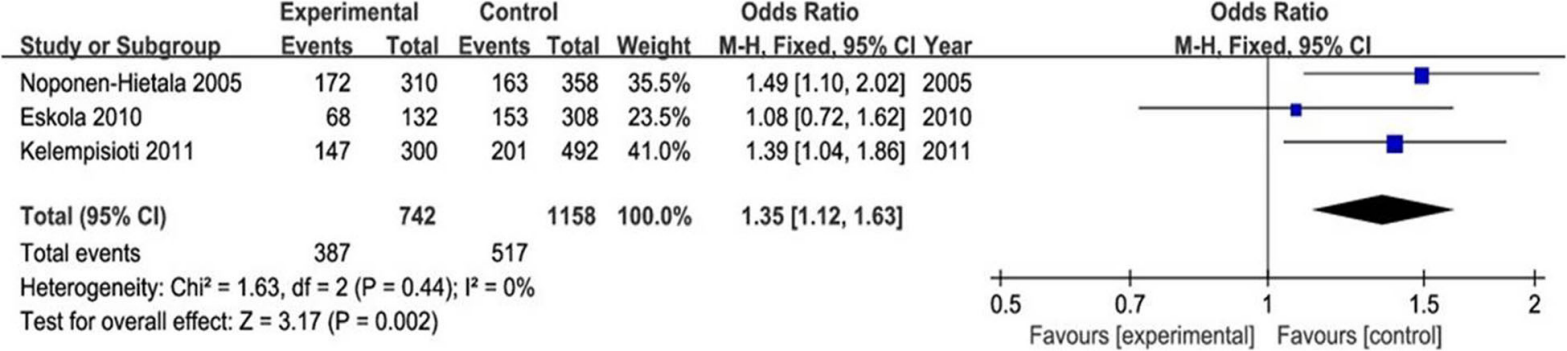
Forest plot of association between IL-6 gene polymorphism (rs1800797) and LDD risk under allelic contract model (G vs. A). There was a significant association between rs1800797 and LDD risk (G vs. A, OR 1.35, 95% CI 1.12–1.63, *P* = 0.002). OR, odds ratio; CI, confidence interval

The meta-analysis of IL-6 and IL-10 gene polymorphisms is presented in Table 3. There was a significant association between IL-6 polymorphisms (rs1800795 and rs1800797) and LDD predisposition in five genetic models (G vs. C, OR 1.39, 95% CI 1.15–1.67, *P* = 0.0005; G vs. A, OR 1.35, 95% CI 1.12–1.63, *P* = 0.002) (Figs. 2 and 3). For subgroup analysis, IL-6 rs1800795 polymorphism was negatively associated with LDD risk in the European population in all genetic models except the dominant model (CC+CG vs. GG). In addition, no significant association of other IL-6 and IL-10 gene polymorphisms (rs1800796, rs13306435, rs2069849, rs1800871, and rs1800896) with LDD was observed (all *P* > 0.05).

**Table 3:** Association test and heterogeneity test of IL-6 and IL-10 gene polymorphisms (rs1800795, rs1800796, rs1800797, rs13306435, rs2069849, rs1800871, and rs1800896)

SNP	Genetic model	Analysis model	Test of association			Heterogeneity test	
			OR	95% Cl	*P* value	*I*^2^	*P*_het_
rs1800795	Allelic						
	G vs. C	Fixed	1.39	[1.15, 1.67]	0.0005	0%	0.77
	Codominant model						
	GG vs.CC	Fixed	1.96	[1.34, 2.86]	0.0005	0%	0.66
	GC vs. CC	Fixed	1.44	[1.04, 1.98]	0.03	0%	0.77
	Dominant model						
	CC+GC vs. GG	Fixed	0.66	[0.48, 0.89]	0.007	0%	0.83
	Recessive model						
	GG+GC vs. CC	Fixed	1.59	[1.17, 2.15]	0.003	0%	0.70
rs1800796	Allelic						
	C vs. G	Random	0.78	[0.55, 1.10]	0.16	63%	0.03
	Codominant model						
	CC vs. GG	Random	0.19	[0.03, 1.06]	0.06	86%	0.0008
	CG vs. GG	Random	0.58	[0.25, 1.32]	0.19	82%	0.0002
	Dominant model						
	GG+CG vs. CC	Random	2.64	[0.61, 11.40]	0.19	96%	< 0.00001
	Recessive model						
	CC+CG vs. GG	Random	0.46	[0.15, 1.42]	0.18	91%	< 0.00001
rs1800797	Allelic						
	G vs. A	Fixed	1.35	[1.12, 1.63]	0.002	0%	0.44
	Codominant model						
	GG vs. AA	Fixed	1.80	[1.23, 2.63]	0.002	0%	0.47
	GA vs. AA	Fixed	1.44	[1.04, 1.98]	0.03	0%	0.68
	Dominant model						
	AA+GA vs. GG	Fixed	0.70	[0.51, 0.95]	0.02	0%	0.56
	Recessive model						
	GG+GA vs. AA	Fixed	1.55	[1.14, 2.10]	0.005	0%	0.58
rs13306435	Allelic						
	A vs. T	Random	2.09	[0.27, 15.87]	0.48	65%	0.09
	Codominant model						
	AA vs. TT	Fixed	NM	NM	NM	NM	NM
	AT vs. TT	Random	2.12	[0.26, 16.97]	0.48	66%	0.09
	Dominant model						
	TT+AT vs. AA	Fixed	NM	NM	NM	NM	NM
	Recessive model						
	AA+AT vs. TT	Random	2.12	[0.26, 16.97]	0.48	66%	0.09
rs2069849	Allelic						
	T vs. C	Fixed	0.52	[0.25, 1.04]	0.07	0%	0.72
	Codominant model						
	TT vs. CC	Fixed	0.36	[0.01, 9.03]	0.54	NM	NM
	TC vs. CC	Fixed	0.55	[0.26, 1.13]	0.10	0%	0.82
	Dominant model						
	CC+TC vs. TT	Fixed	2.61	[0.11, 64.62]	0.56	NM	NM
	Recessive model						
	TT+TC vs. CC	Fixed	0.52	[0.25, 1.08]	0.08	0%	0.76
rs1800871	Allelic						
	A vs. C	Random	1.11	[0.63, 1.95]	0.72	91%	0.0008
	Codominant model						
	AA vs. CC	Random	1.10	[0.40, 2.99]	0.85	87%	0.006
	AC vs. CC	Fixed	0.92	[0.65, 1.30]	0.65	0%	0.49
	Dominant model						
	CC+AC vs. AA	Random	0.84	[0.38, 1.88]	0.68	91%	0.0008
	Recessive model						
	AA+AC vs. CC	Random	1.02	[0.56, 1.86]	0.95	69%	0.07
rs1800896	Allelic						
	G vs. A	Random	1.2	[0.64, 2.88]	0.57	92%	< 0.00001
	Codominant model						
	GG vs. AA	Random	0.79	[0.41, 1.50]	0.47	67%	0.08
	GA vs. AA	Random	1.28	[0.58, 2.97]	0.54	89%	0.0002
	Dominant model						
	AA+GA vs. GG	Fixed	1.25	[0.95, 1.66]	0.11	50%	0.16
	Recessive model						
	GG+GA vs. AA	Random	1.23	[0.53, 2.83]	0.63	91%	< 0.0001

*NM* not mentioned, *SNP* single nucleotide polymorphisms, *OR* indicates odds ratio, *CI* indicates confidence interval, *P*_*het*_ indicates *P* value for heterogeneity

**Fig. 4:**
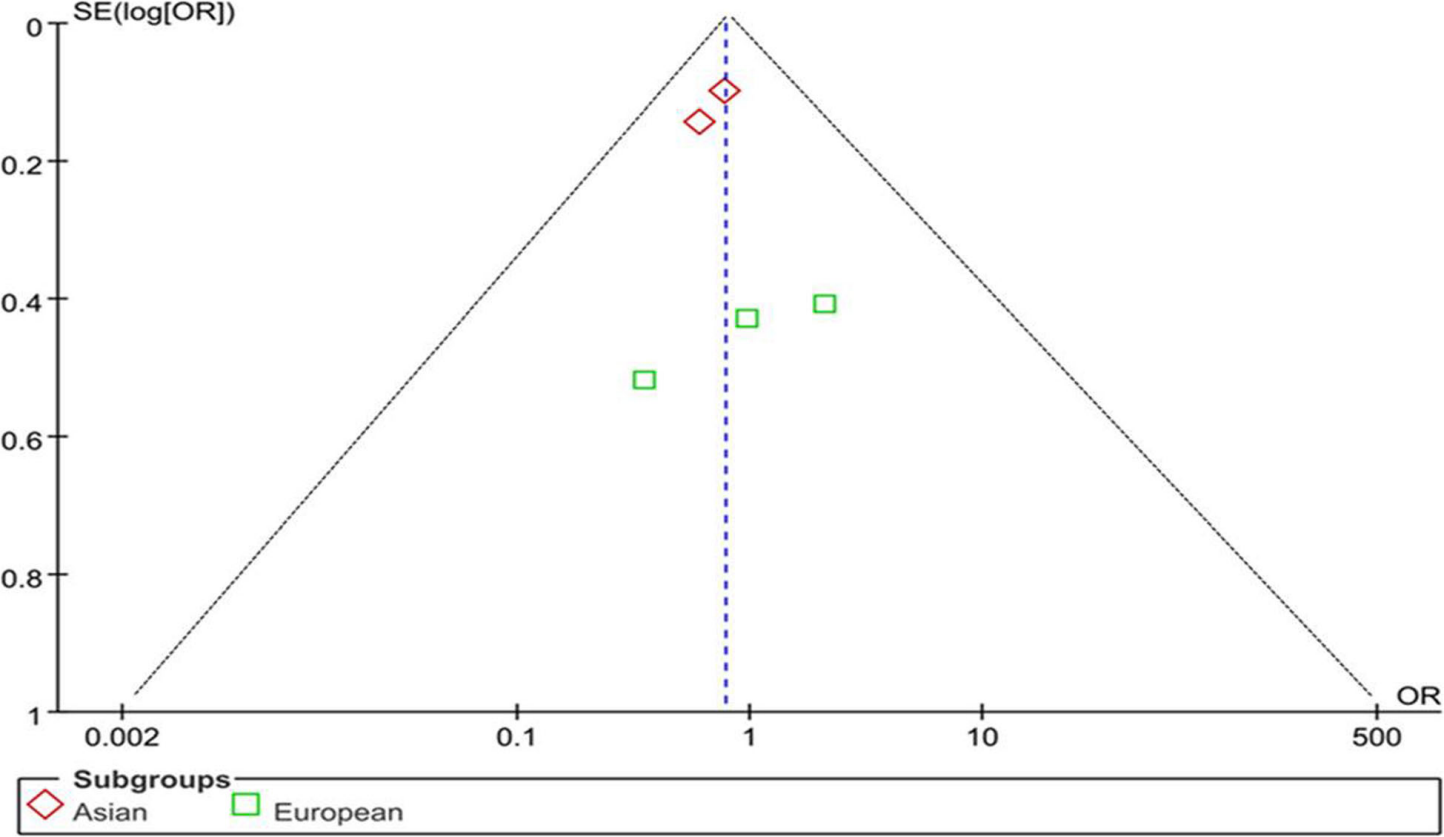
Funnel plot analysis for publication bias in selection of studies on IL-6 gene polymorphism (rs1800796) under allelic contract model (C vs. G). There was no significant publication bias (*P* > 0.05)

Sensitivity analysis was performed by excluding one study at a time to analyze the heterogeneity. For rs1800896, the overall results were not altered in all genetic models after omitting the study by Huang et al. [14], and the heterogeneity was obviously reduced after omitting Huang’s study (Table 4). For rs1800796, the result of the allelic contrast model suggested a negative association between rs1800796 and LDD after excluding the study reported by Eskola et al. [11], and the heterogeneity was significantly reduced after excluding Eskola’s study (Table 5). According to Begg’s funnel plot and Egger’s regression tests, the result indicated no significant publication bias under all genetic models (all *P* > 0.05 for all models tested) (Fig. 4).

**Table 4:** Heterogeneity analysis and Egger regression analysis IL-6 and IL-10 gene polymorphisms (rs1800795, rs1800796, rs1800797, rs13306435, rs2069849, rs1800871, and rs1800896)

SNP	Analysis model	Heterogeneity analysis			Egger regression analysis		
		*χ*^2^	*P*	*I*^2^ (%)	*t*	95% CI	*P*
rs1800795	Fixed	1.14	0.77	0	− 1.54	[− 3.58, 1.69]	0.263
rs1800796	Random	10.89	0.03	63	0.37	[− 4.15, 5.24]	0.736
rs1800797	Fixed	1.63	0.44	0	− 4.19	[− 14.66, 7.38]	0.149
rs13306435	Random	2.84	0.09	65			
rs2069849	Fixed	0.13	0.72	0			
rs1800871	Random	11.17	0.0008	91			
rs1800896	Random	24.97	< 0.00001	92	14.47	[1.52, 23.42]	0.044

**Table 5:** The result of sensitivity analysis with each study omitted for rs1800796 in IL-6 gene and rs1800896 in IL-10 gene

Study omitted	OR	95% CI	*P*
rs1800796 C/G			
Noponen-Hietala [10]	0.84	[0.59, 1.19]	0.32
Eskola [11]	0.70	[0.56, 0.87]	0.001
Kelempisioti [12]	0.76	[0.52, 1.12]	0.17
Huang [14]	0.89	[0.51, 1.55]	0.67
Zhu [15]	0.82	[0.43, 1.56]	0.55
rs1800896 G/A			
Noponen-Hietala [10]	1.30	[0.39, 4.33]	0.67
Lin [13]	1.59	[0.71, 3.57]	0.26
Huang [14]	0.86	[0.58, 1.27]	0.46

*OR* indicates odds ratio, *CI* confidence interval

### IL-6 and IL-10 expressions and LDD

**Fig. 5:**
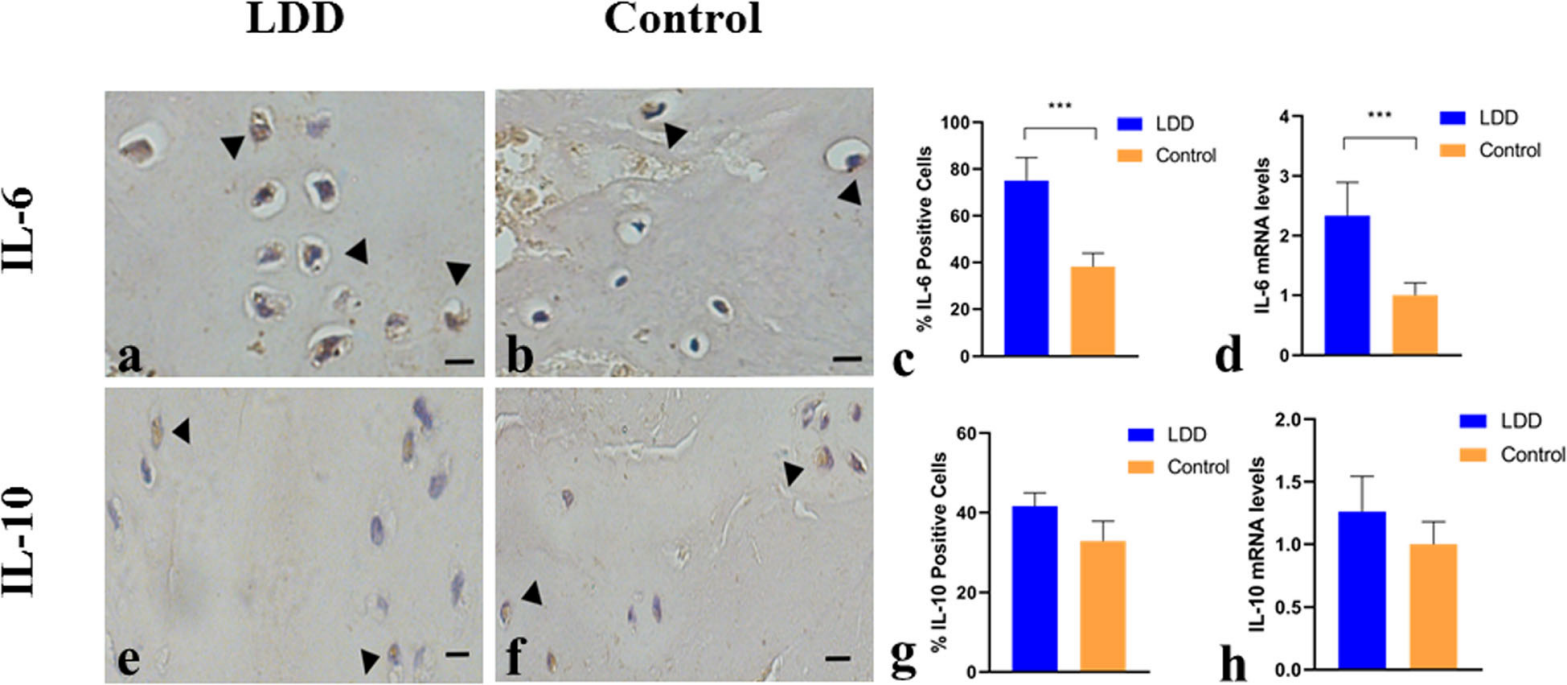
The expression levels of IL-6 and IL-10 in the LDD group and the control group. The positive signals were identified in the cytoplasm of nucleus pulpous cells in the LDD group (**a**, **e**) and the control group (**b**, **f**) (magnification, × 400). The black arrowheads show IL-6 and IL-10 positive cells. Bar charts (**c**, **g**) show the expression levels of IL-6 and IL-10 in the LDD group and the control group. Bar charts (**e**, **h**) show the mRNA levels of IL-6 and IL-10 in the LDD group and the control group. Results are mean ± SD. ***P < 0.001

The proteins of IL-6 and IL-10 expressed in the cytoplasm were detected in the nucleus pulpous of the intervertebral disc. Compared with the control group, the LDD group had significantly higher expression of IL-6 (IL-6 immunopositive cells, 73.68 ± 10.99% vs. 37.23 ± 6.42%; *P* < 0.001). However, there were no significant differences in IL-10 expression between the two groups (IL-10 immunopositive cells, 41.18 ± 23.56% vs. 32.79 ± 20.56%; *P* = 0.325). For RT-PCR, there were higher IL-6 mRNA levels in the LDD group than those in the control group (*P* < 0.001). No significant differences in IL-10 expression levels were found in the two groups (*P* = 0.112) (Fig. 5).

## Discussion

Rigal and colleagues [25] initially used a meta-analysis to validate the effects of genetic polymorphisms on disc degeneration and demonstrated that IL-6 rs1800797 polymorphism was identified as a positive gene locus of LDD. Recently, several studies were published to explicate the association between IL-6 and IL-10 gene polymorphisms and LDD risk [14, 15]. Larger sample size of meta-analysis was essential to examine reliability and accuracy of the conclusion [26]. Hence, a comprehensive meta-analysis needs to update with data from the latest studies. The current study, including 1456 cases and 1611 controls, showed that two IL-6 gene polymorphisms (rs1800795 and rs1800797) were significantly associated with susceptibility to LDD in all genetic models. In accordance with Northern European reports [11, 12], our study identified that the G allele represented approximately 1.38- and 1.35-fold increased risk factor for developing disc degeneration. Furthermore, we performed the subgroup analysis stratified by ethnicity. After subgroup analysis, it revealed that the IL-6 rs1800795 polymorphism is associated with LDD in European population but not in Asian population. The heterogeneity of our study should be observed in interpreting the result. Various study designs and genetic background may be explicated the main causes of heterogeneity. More importantly, the presence of heterogeneity may result from the different diagnostic criteria of LDD and phenotype selection [27, 28]. There are obvious discrepancies in the clinical phenotype of LDD patients, such as annular tears, disc herniation, spinal stenosis, and spondylolisthesis. We could postulate that various phenotypes might represent different disease courses of LDD, which may be influenced by variable genetic polymorphisms [29].

IL-6 is a pro-inflammatory cytokine which plays a vital role in the regulation of host immune response in intervertebral disc [30]. We performed an in silico analysis for evaluating the possible functional implication of rs1800795 and rs1800797 polymorphism by using rSNPBase (http://rsnp3.psych.ac.cn/) [31] and found that these two SNPs were located within the promoter of IL-6 gene, which had a possible transcriptional regulatory effect. The G to C polymorphism at position-174 of the IL-6 gene (rs1800795) causes differential activity in promoter constructs which upregulates IL-6 gene transcription [32]. The G allele of rs1800795 promotes higher circulating levels of IL-6 in patients with sepsis [33]. However, the relationship between IL-6 polymorphisms and IL-6 expression in LDD patients has not been reported. Our meta-analysis confirmed that IL-6 gene polymorphisms were significantly associated with LDD, and the G allele of rs1800795 and rs1800797 represented the risk factors for LDD. Furthermore, the results of IHC and RT-PCR analysis showed that increased IL-6 expression levels were found in the degenerated disc. Compared with GC/CC or GA/AA genotypes, GG genotypes of rs1800795 and rs1800797 were associated with higher levels of IL-6 expression. Thus, we postulated that rs1800795 GG or/and rs1800797 GG genotypes were the genetic risk factors for progression of LDD, probably by decreasing the expression of IL-6. IL-6 rs1800795 and rs1800797 polymorphisms, located within regulatory regions, could be part of RNA-binding protein site and could be involved in RNA-binding which is protein-mediated. Previous studies showed that IL-6 significantly induced disc degeneration by activating STAT3 and β-catenin signaling pathways [30, 34]. It seems to indicate that IL-6 might act synergistically with other genetic factors contributing to the risk of disc degeneration.

Rs13306435 and rs2069849 were located in exon 5 of IL-6 gene. The T>A variation of rs13306435 changed an amino acid from Asp to Glu. The SNP rs2069849 (C>T) was a synonymous variant. The T allele of 13306435 and the C allele of rs2069849 had been reported previously to be associated with increased expression and plasma levels of IL-6 [32]. However, other studies did not found any association [35, 36]. Karppinen et al. [37] supposed that these two SNPs were unlikely to cause disease but may be in linkage disequilibrium with the functional mutation some distance away from it. In addition, rs1800871 and rs1800896 were two promoter polymorphisms of IL-10, which may lead to alteration of the specific transcription factor recognition sites [38]. Gibson et al. found that two SNPs were not associated with the differential IL-10 expression in LDD, and other polymorphisms, particularly those in the distal part of the promoter, may have an effect on IL-10 production [39]. Based on recent research, the effects of IL-10 promoter polymorphisms on gene expression are likely to be more complex than what has been initially expected [40].

Several limitations of this study should be acknowledged. First, heterogeneity could result from different phenotype selection and diagnostic criteria of LDD. It may exert an influence on the reliability of meta-analysis. Second, we did not perform the subgroup analysis stratified by gender, age, and environmental factors as the original data were unavailable. Third, for IHC and RT-PCR studies, the sample size was relatively small, which may increase the risk of false positive. More evidence is needed to validate these associations.

## Conclusions

Our study indicated that IL-6 gene polymorphisms (rs1800795 and rs1800797) were significantly associated with susceptibility to LDD. IL-6 expression levels may be may be an important risk factor for LDD.

## Data Availability

Please contact the authors for data requests.

## Abbreviations

IL-10: Interleukin-10
IL-6: Interleukin-6
LBP: Low back pain
LDD: Lumbar disc degeneration
SNP: Single nucleotide polymorphism
